# Granulomatous secondary syphilis with pulmonary involvement

**DOI:** 10.1016/j.jdcr.2023.11.017

**Published:** 2023-12-02

**Authors:** Andrew D. Johnston, Simon F. Roy, Jennifer McNiff, Marco Petrazzuoli

**Affiliations:** a Department of Dermatology, Yale School of Medicine, New Haven, Connecticut; b Department of Dermatology, West Haven VA Medical Center, West Haven, Connecticut

**Keywords:** granulomatous syphilis, HIV, human immunodeficiency virus, otologic syphilis, pulmonary syphilis, secondary syphilis, secondary syphilis with otologic involvement, secondary syphilis with pulmonary involvement

## Introduction

*Treponema pallidum*, the causative agent of syphilis, is a spirochete that can only be cultured *in vivo* and has various clinical morphologies, leading to diagnostic difficulty. We describe a rare case of granulomatous secondary syphilis with pulmonary and otologic involvement in the setting of human immunodeficiency virus (HIV)-positivity.

## Case Report

We present the case of a 65-year-old man who has sex with men and has prior history of treated syphilis and HIV (CD4 >600 cells/mm^3^; viral load undetectable). Due to an extensive smoking history, he underwent a screening low-dose lung computed tomography, which found two >2 cm spiculated masses with multiple satellites in his right lower lobe. To work-up malignancy, a positron emission tomography-computed tomography was performed and exhibited innumerable nodules in the lower portion of the lungs. The rapid change in imaging was concerning for an infectious etiology and prompted an inpatient admission for expedited work-up. The patient described a nonproductive cough and an asymptomatic, cephalocaudal rash.

**Fig 1:**
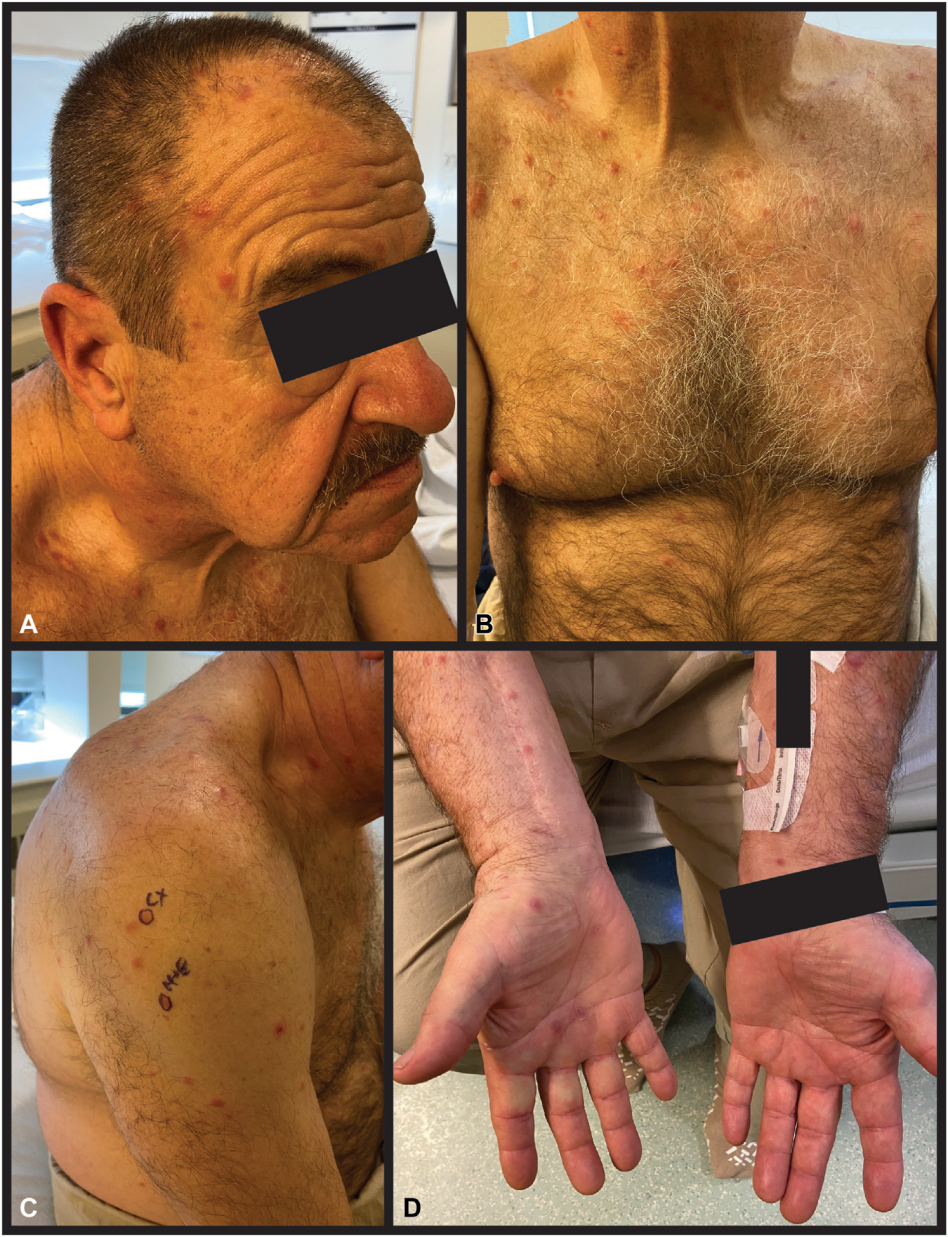
On the head **(A)**, upper chest **(B)**, and upper portion of the left arm **(C)**, there are representative 5 to 10 mm firm, nontender pink papulonodules without secondary change, which were also noted on the back and legs (not shown). The skin biopsy site is denoted as “H+E” in panel **(C)**. The right palm has 4 ∼5 mm round, thin pink-brown papules with annular scale, consistent with “copper pennies” **(D)**.

On examination, there were dozens of diffusely scattered 5 to 10 mm firm, nontender pink papulonodules without surface changes favoring the head, upper torso, and upper extremities (Fig 1, *A*-*C*). The palmar surfaces were noted to have several ∼5 mm round, thin pink-brown papules with annular scale, consistent with “copper pennies” (Fig 1, *D*). The differential diagnosis of his lesions included secondary syphilis, cutaneous metastases (with likely lung primary), lymphomatoid papulosis, sarcoidosis or other histiocytic disorder, and mycobacterial or dimorphic fungal infection.

**Fig 2:**
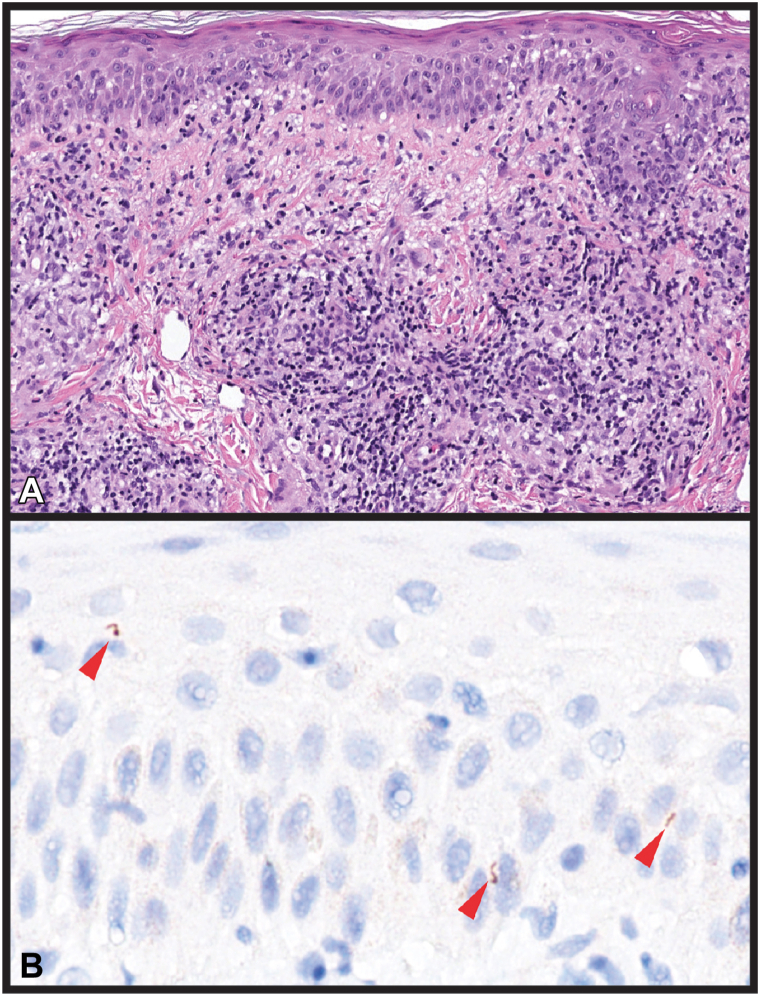
**A,** Hematoxylin and eosin stain demonstrates ill-formed granulomas with lymphocytic and plasma cell inflammation throughout the dermis. **B,** Immunohistochemistry toward spirochete antigen with background hematoxylin reveals few spindled organisms (brown, denoted by *red arrows*) within intercellular spaces between keratinocytes in the stratum basale and spinosum.

An extensive infectious work-up, including a sterile skin biopsy culture, was initiated. A biopsy from the R upper portion of the arm demonstrated ill-formed granulomas with a mixed inflammatory infiltrate, including plasma cells (Fig 2, *A*); immunohistochemistry of spirochete antigen led to the diagnosis of syphilis, yet only revealed a few scattered organisms (Fig 2, *B*). Subsequently, the patient’s fluorescent treponemal antibody absorbed test resulted positive, and his positive rapid plasma reagin titer was determined to be 1:16. Further questioning revealed subjective hearing loss, in the absence of other neurologic symptoms. Because isolated auditory symptoms are likely to have normal cerebrospinal fluid findings, and the treatment regimen is the same as neurosyphilis, a lumbar puncture was not pursued in concordance with current recommendations.^1^ The case was reported to the Centers for Disease Control and Prevention, and the patient received 2.4 million units of penicillin-G intramuscularly followed by continuous intravenous infusion of 18 million units per day for 14 days.

Three weeks after finishing treatment course, a repeat chest computed tomography confirmed radiographic response of lung involvement to antisyphilitic therapy. Despite significant improvement on examination, 2 papulonodules remained on the left forearm—the first lesions noticed by the patient. A biopsy demonstrated a similar, although more inflamed, granulomatous pattern with fragments of spirochete-stain positive organisms. Therefore, the patient received 3 more doses of 2.4 million units penicillin-G every week to achieve complete resolution.

## Discussion

Since the start of the COVID-19 pandemic, the number of syphilis cases has been on the rise in United States, as evidenced by a 28.6% surge in primary and secondary cases from 2020 to 2021.^2^ Men who have sex with men are disproportionately impacted, accounting for 46.5% of all male primary and secondary cases in 2021. They also have the highest prevalence of HIV coinfection (47%), compared with men who have sex with women only (10.7%).^3^ Our patient had these additional risk factors and highlights the need for increased vigilance for atypical presentations of syphilis, “the great mimicker” in these “at-risk” patient populations.

Pulmonary involvement in secondary syphilis is exceedingly rare with only 41 cases reported in English. Fortunately, our patient’s pulmonary nodules copresented with a rash, which was also observed in 32 of the 41 previously reported cases (Supplementary Table I, available via Mendeley at https://data.mendeley.com/datasets/2cw567k73r/1). This allowed for immunohistochemistry of easily obtained formalin-fixed tissue from skin, which has largely replaced dark field microscopy. In the absence of skin findings or to confirm lung involvement, transbronchial biopsy, bronchial aspirate, or computed tomography-guided percutaneous needle aspiration can be performed, followed by immunohistochemistry or polymerase chain reaction.^4^ More recently, bronchoalveolar lavage followed by metagenomic next generation sequencing has been utilized to diagnose pulmonary syphilis.^5^

Although the patient demonstrated classic palmar involvement with “copper pennies,” his primary lesions were pink papulonodules without secondary change and histologically exhibited ill-formed granulomas. This is an uncommon presentation of syphilis, with around 44 individual cases reported in the English literature (Supplementary Table II, available via Mendeley at https://data.mendeley.com/datasets/2cw567k73r/1). Contrary to our patient, the majority (16/23) of reviewed granulomatous syphilis cases in Rysgaard et al^6^ did not have palmoplantar involvement. Therefore, the absence of these “classic” lesions does not exclude a diagnosis of syphilis, and a patient may present with multiple morphologies.

There is no established reason as to why a patient will develop granulomas in response to syphilis, but there are 2 main hypotheses: (1) a late stage of secondary syphilis, correlating with longer disease duration and (2) a type IV hypersensitivity-like reaction primed by a prior infection with syphilis. Evidence for the first hypothesis comes from a retrospective review of 64 skin biopsies of secondary syphilis, in which all but one sample obtained >16 weeks after rash presentation demonstrated granulomatous features.^7^ However, the same study notes that 2 specimens from lesions of only 4 week duration had focal granulomas,^7^ and as in our patient, an initial papular and/or nodular presentation was seen in 22 of 32 cases reviewed in Rysgaard et al.^6^ Also disfavoring this hypothesis, a more recent review of 8 primary and 26 secondary syphilis biopsies found granulomatous inflammation in 37% and 38%, respectively.^8^ The second hypothesis stems from a 1965 study that found 10 of 26 prisoners, with known prior syphilis, had granulomatous skin lesions after treponemal inoculation.^9^ Even though most case studies do not provide prior syphilis history, 6 of the aforementioned 44 cases, as well as our patient, had a known prior syphilis infection (Supplementary Table II).

Although our patient did not undergo pulmonary biopsy, we observed the resolution of his radiographic findings after treatment, and speculate that the pathology was likely granulomatous, given the 2 >2 cm nodules. Govender et al^4^ found that 5 of 10 pulmonary syphilis cases with lung biopsies had granulomatous pathology. There may also be a correlation with HIV coinfection and pulmonary involvement, since 10 (34%) of 29 cases with pulmonary involvement were HIV positive upon testing (Supplementary Table I). This association is lower in granulomatous syphilis; of the 26 reported cases, in which HIV status was mentioned, only 5 were positive (19%; Supplementary Table II). These high proportions of rare manifestations may be due to the high prevalence of HIV and syphilis coinfection, or reflect an ineffective immune response to syphilis, despite the vast majority of these patients having normal CD4+ cell counts. Additionally, the immune dysregulation caused by HIV may lead to a propensity for granuloma formation, as evidenced by HIV-associated granuloma annulare.^10^ Regardless of causation or correlation, there should be a higher suspicion for atypical presentations of syphilis in HIV-positive patients.

With the rise of syphilis cases, we need to familiarize ourselves with less common presentations, such as granulomatous secondary syphilis with pulmonary involvement.

## Conflicts of interest

None disclosed.

## References

[1] Centers for Disease Control and Prevention (2021).

[2] Centers for Disease Control and Prevention (2021).

[3] Kidd S., Torrone E., Su J., Weinstock H. (2018). Reported Primary and Secondary Syphilis Cases in the United States: implications for HIV infection. Sex Transm Dis.

[4] Govender D., Jackson C., Chetty D. (2021). Syphilitic pulmonary inflammatory pseudotumor: a diagnostic challenge. Int J Surg Pathol.

[5] Yang X., Wu W., Wang Y., Wu W., Huang X., Xu L. (2022). A case of secondary pulmonary syphilis – the utility of mNGS in bronchoalveolar lavage fluid: a case report. Infect Drug Resist.

[6] Rysgaard C., Alexander E., Swick B.L. (2014). Nodular secondary syphilis with associated granulomatous inflammation: case report and literature review. J Cutan Pathol.

[7] Abell E., Marks R., Jones E.W. (1975). Secondary syphilis: a clinico-pathological review. Br J Dermatol.

[8] Martín-Ezquerra G., Fernandez-Casado A., Barco D. (2009). *Treponema pallidum* distribution patterns in mucocutaneous lesions of primary and secondary syphilis: an immunohistochemical and ultrastructural study. Hum Pathol.

[9] Magnuson H.J., Thomas E.W., Olansky S., Kaplan B.I., De Mello L., Cutler J.C. (1956). Inoculation syphilis in human volunteers. Medicine.

[10] Toro J.R., Chu P., Yen T.S.B., LeBoit P.E. (1999). Granuloma annulare and human immunodeficiency virus infection. Arch Dermatol.

